# Comparative Untargeted Metabolomics Analysis of the Psychostimulants 3,4-Methylenedioxy-Methamphetamine (MDMA), Amphetamine, and the Novel Psychoactive Substance Mephedrone after Controlled Drug Administration to Humans

**DOI:** 10.3390/metabo10080306

**Published:** 2020-07-27

**Authors:** Andrea E. Steuer, Daria Kaelin, Martina I. Boxler, Lisa Eisenbeiss, Friederike Holze, Patrick Vizeli, Joanna Czerwinska, Paul I. Dargan, Vincenzo Abbate, Matthias E. Liechti, Thomas Kraemer

**Affiliations:** 1 Department of Forensic Pharmacology & Toxicology, Zurich Institute of Forensic Medicine, University of Zurich, 8057 Zurich, Switzerland; kaelinda@student.ethz.ch (D.K.); martina.boxler@irm.uzh.ch (M.I.B.); lisa.eisenbeiss@irm.uzh.ch (L.E.); thomas.kraemer@irm.uzh.ch (T.K.); 2 Department of Biomedicine and Department of Clinical Research, Division of Clinical Pharmacology and Toxicology, University Hospital Basel, University of Basel, 4056 Basel, Switzerland; friederike.holze@usb.ch (F.H.); patrick.vizeli@usb.ch (P.V.); matthias.liechti@usb.ch (M.E.L.); 3 King’s Forensics, Department of Analytical, Environmental and Forensic Sciences, King’s College London, London WC2R 2LS, UK; joanna.czerwinska@kcl.ac.uk (J.C.); vincenzo.abbate@kcl.ac.uk (V.A.); 4 Clinical Toxicology, Guy’s and St Thomas’ NHS Foundation NHS Trust, London SE1 9RT, UK; Paul.Dargan@gstt.nhs.uk; 5 Clinical Toxicology, Faculty of Life Sciences and Medicine, King’s College London, London WC2R 2LS, UK

**Keywords:** psychoactive stimulants, untargeted metabolomics, mephedrone, amphetamine, MDMA, LC-HRMS

## Abstract

Psychoactive stimulants are a popular drug class which are used recreationally. Over the last decade, large numbers of new psychoactive substances (NPS) have entered the drug market and these pose a worldwide problem to human health. Metabolomics approaches are useful tools for simultaneous detection of endogenous metabolites affected by drug use. They allow identification of pathways or characteristic metabolites, which might support the understanding of pharmacological actions or act as indirect biomarkers of consumption behavior or analytical detectability. Herein, we performed a comparative metabolic profiling of three psychoactive stimulant drugs 3,4-methylenedioxymethamphetamine (MDMA), amphetamine and the NPS mephedrone by liquid chromatography-high resolution mass spectrometry (LC-HRMS) in order to identify common pathways or compounds. Plasma samples were obtained from controlled administration studies to humans. Various metabolites were identified as increased or decreased based on drug intake, mainly belonging to energy metabolism, steroid biosynthesis and amino acids. Linoleic acid and pregnenolone-sulfate changed similarly in response to intake of all drugs. Overall, mephedrone produced a profile more similar to that of amphetamine than MDMA in terms of affected energy metabolism. These data can provide the basis for further in-depth targeted metabolome studies on pharmacological actions and search for biomarkers of drug use.

## 1. Introduction

**Figure 1:**
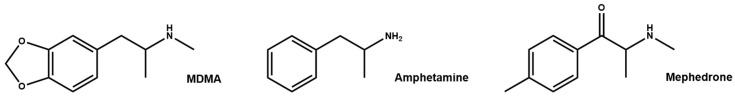
Chemical formulas of the psychostimulants 3,4-methylenedioxymethamphetamine (MDMA), amphetamine, and mephedrone.

Drugs of abuse (DOA) consumption remains a worldwide threat to human health, particularly with large numbers of new psychoactive substances (NPS) emerging each year. By the end of 2018, more than 700 NPS were being monitored by the European Monitoring Centre for Drugs and Drug Addiction (EMCDDA), with 55 newly reported in 2018. About one third of the NPS reported in Europe belong to the class of psychostimulants [1]. Psychoactive stimulants are a particularly popular class of drugs which are used recreationally. Based on their pharmacological action, stimulants can be further divided into classic pure stimulants such as amphetamine and methamphetamine and more empathogenic/entactogenic acting drugs like the prototypical entactogen 3,4-methylenedioxymethamphetamine (MDMA) [2,3,4,5]. Mephedrone, chemically a synthetic cathinone, was one of the first described NPS stimulants in 2007, and has been shown to exhibit psychostimulant, entactogenic and hallucinogenic effects [6,7,8]. Amphetamine, MDMA and mephedrone (chemical structures given in Figure 1) share similar pharmacological properties by acting as indirect monoaminergic agonists through the release of different neurotransmitters and/or the inhibition of their respective reuptake transporter in the presynaptic membrane. Amphetamine rapidly releases dopamine (DA) and norepinephrine (NE), while MDMA is primarily a releaser of serotonin (5-hydroxytryptamine, 5-HT) and oxytocin [3,9,10]. Mephedrone’s mode of action resembles this of both compounds—MDMA and amphetamine—with rapid releasing properties for DA and 5-HT [6,11]. For newer (psychostimulant) NPS, information on their pharmacological action, adverse effects, toxicity, pharmacokinetics and detectability in humans is typically scarce, at least immediately after their first appearance [12]. Many mephedrone-like compounds have appeared on the market and have been shown to exhibit relatively diverse in vitro pharmacological properties either more similar to amphetamine or to MDMA [11,13,14,15]. However, data on their in vivo pharmacological behavior as well as possibilities for their rapid detectability in biological samples are certainly needed for risk assessment and clinical and forensic toxicological investigations. Full pharmacological evaluation of each NPS seems unfeasible, being too work-intensive and thereby time-consuming to guarantee risk assessment and health prevention in reasonable time [16]. In addition, their direct detection and identification remains an analytical challenge due to their rapid transience and the (initial) lack of certified reference material [12,16,17].

Metabolomic approaches represent interesting tools for the simultaneous, (high-throughput) detection of many endogenous metabolites within single samples. They typically involve both the identification of endogenous substances present in biological samples and the elucidation of statistical differences between two or more conditions such as drug intake. At best, several pathways or even characteristic metabolites could be identified, even for otherwise pharmacologically uncharacterized NPS, which might support the understanding of pharmacological and toxicological actions or act as indirect biomarkers of consumption behavior or analytical detectability [12,16,18]. For instance, Olesti et al. described a novel approach that successfully applied targeted metabolomics to predict pharmacological profiles of NPS in rat plasma, urine and different brain areas. Metabolic profiles of mephedrone and JWH-018 were compared to those of commonly known drugs including amphetamine, MDMA and tetrahydrocannabinol (THC) [16]. However, targeted analysis was performed on neurotransmitters and steroids only. Untargeted metabolome analysis might also reveal other pathways and might be linked to pharmacological action and comparison among drugs. The metabolome is highly dynamic and sensitive to many confounding factors, such as stress, food intake or physical exercise [19,20]. To exclude confounding factors and to trace observed metabolic changes directly back to drug intake, highly controlled study conditions are necessary. Only few studies on drugs of abuse and particularly NPS have been performed until now [12,16,21], the majority of them in rodents. Most valuable (controlled) human data are limited due to ethical restrictions and so far only available for MDMA [22,23,24], cocaine [25,26], and gamma-hydroxybutyric acid (GHB) [27,28,29].

The aim of the present study was to apply untargeted metabolomics profiling on plasma samples from controlled drug administration studies to humans to investigate (a) the changes in the human metabolome caused by the psychostimulants amphetamine and mephedrone, (b) to compare the influence of the psychostimulants drugs (MDMA [24], amphetamine and mephedrone) on the human metabolome, and (c) to identify biological pathways affected by the intake of psychostimulants which would be valuable for further targeted analysis.

## 2. Results

### 2.1. Analytical and Data Processing Procedures of Samples from Controlled Clinical Studies

The plasma samples selected for the presented comparative, metabolic profiling study with the three psychostimulant drugs MDMA, amphetamine and mephedrone were obtained from former clinical studies initially designed for investigations of pharmacological effects [5,30] and pharmacokinetic parameters (mephedrone, unpublished data). All studies performed plasma sampling prior to drug administration (t0), and MDMA and amphetamine studies additionally included a placebo session (crossover design). Post-dose plasma samples for reanalysis were available for 16 (MDMA), 13 (amphetamine), and 5 (mephedrone) participants at the available time-points during the time of maximum plasma concentration (tmax) (4 h for MDMA, 3 h for amphetamine, and mephedrone) and time of declining plasma concentrations (8 h, 7.5 h, 6 h for MDMA, amphetamine, and mephedrone, respectively).

We used a more or less state-of-the-art untargeted metabolomics approach applying both reversed-phase (RP) and hydrophilic liquid interaction chromatography (HILIC) with positive (pos) and negative (neg) polarity to measure the abundance of hundreds of metabolites with a broad range of different physicochemical properties at high mass accuracy [31]. MS only and additional MS/MS measurements were performed on a triple quadrupole time of flight (Q-TOF) instrument. System suitability tests (SST) and pooled plasma quality control (QC) samples were regularly measured to monitor analytical instrument performance. Additionally, all samples were measured in randomized order within each batch. Mean coefficients of variations (%CV) values of all compounds in the SST mix in RP pos, RP neg, HILIC pos and HILIC neg were 8%, 16%, 13%, and 13%, respectively in the amphetamine batches and 8%, 21%, 14%, and 9%, respectively in the mephedrone batches. Median %CV of all features in pooled samples in RP pos, RP neg, HILIC pos and HILIC neg were 16%, 17%, 23%, and 28%, respectively for amphetamine batches and 7%, 9%, 13%, and 15%, respectively for mephedrone batches and fulfilled the acceptance criteria.

Following MS measurements, each batch was loaded into Progenesis Qi separately for retention time (RT) alignment, peak picking and data deconvolution (grouping of adducts of the same compounds). Finally, a list of all extractable features (defined by its RT and accurate mass) within one batch were exported and a series of statistical analysis applied to identify plasma metabolites that are significantly affected by one or multiple substances. The highly controlled study conditions (pre-dose sample, crossover design) allowed for paired statistical testing for each drug, beneficial to account for the expected and observed high inter-individual variations. In addition to classic univariate analysis (paired t-test, raw *p*-values, *p* < 0.05) combined with fold-change (fc) analysis (general fc > 1.5), a mixed-effect model in R studio with increased statistical power was applied to the amphetamine set.

### 2.2. Effects of MDMA on the Human Metabolome

The influence of MDMA on the human plasma metabolome has previously been published elsewhere [24]. Briefly, mainly amino acids, phospholipids and steroids were altered after consumption of MDMA. Some compounds belonging to the class of acyl carnitines and fatty acids have also been affected by MDMA. As expected, the best discrimination power was achieved by MDMA itself or its known metabolites.

### 2.3. Effects of Amphetamine on the Human Metabolome

Amphetamine itself was undetectable in the current data set, even when a targeted search was applied on the untargeted acquired data. This was due to the lack of sensitivity of the Q-TOF instrument and the untargeted acquisition (data not shown). Amphetamine plasma concentrations in the initial clinical study following the 40 mg dose have been published elsewhere using targeted methods and reached a maximum plasma concentration (Cmax) of 68–133 (mean 100) ng/mL 2.6 (1.0–5.5) h after intake [5].

**Figure 2:**
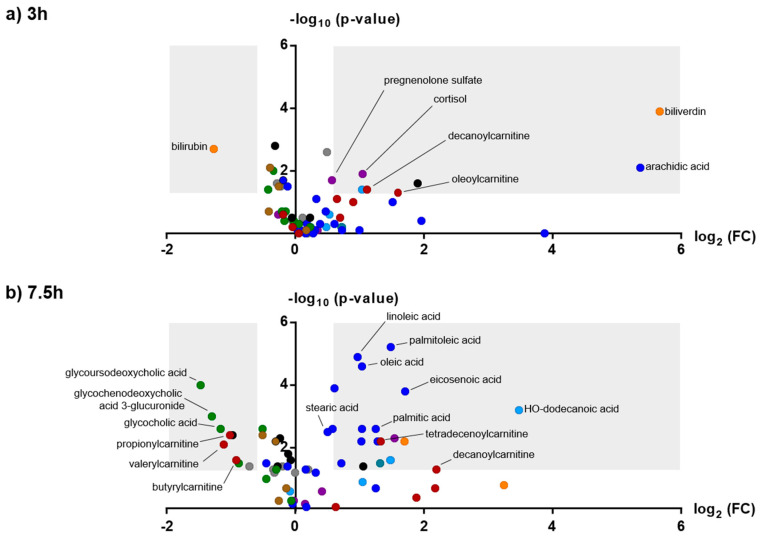
Volcano plots of selected, identified features 3 h (**a**) and 7.5 h (**b**) after oral amphetamine intake. Different compounds classes are depicted in different colors: carnitines (red), long-chain fatty acids (dark blue), medium-chain/short-chain fatty acids (light blue), bile acids (green), amino acids (brown), phospholipids (grey) and biliverdin/bilirubin (orange). All other features remained black. Compounds in light grey areas can be considered as particularly interesting (−log10 *p*-value > 1.3; log2 foldchange > 0.6 or < −0.6).

**Figure 3:**
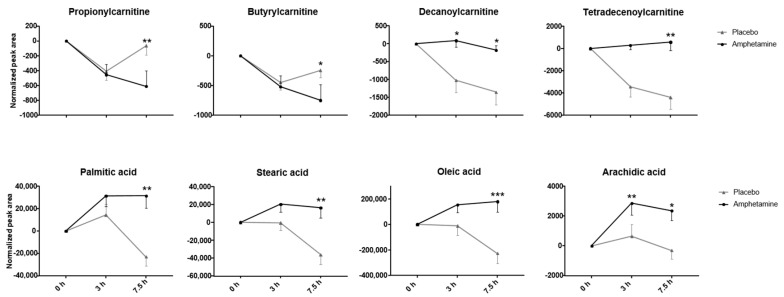
Influence of amphetamine on different short- and medium-chain acyl carnitines (upper panel) and long-chain fatty acids (lower panel). Black lines indicate drug, light grey lines placebo intake, respectively. Data points represent mean and standard error of mean (SEM) of 13 replicates. Statistical comparison was performed between placebo and drug intake using paired *t*-tests (*p* < 0.05; n.s. > 0.05; * 0.01–0.05; ** 0.001–0.01; *** 0.0001–0.001).

With the combination of four different analytical methods (RP pos/neg and HILIC pos/neg), 11,108 features were extracted after data deconvolution in Progenesis Qi. After setting the criteria of abundance greater than 500 counts per seconds (cps) in more than 10 samples, 3981 features remained for statistical analysis. Univariate analysis (paired t-test, *p* < 0.05; fc > 1.5) returned 259 potentially interesting features and the mixed effect model (raw *p*-values < 0.05) indicated 225 affected features 3 h or 7.5 h after amphetamine intake, respectively. In total, 57 compounds could be identified with a confidence level of 1 or 2 according to the Metabolomics Standard Initiative (MSI) [32] and these are summarized in Table S1 of the Supplementary Information. Respective volcano plots 3 h and 7.5 h after amphetamine intake are depicted in Figure 2. They mainly belong to the compound classes of acyl carnitines, amino acids, bile acids, short-, medium- and long-term fatty acids, and steroids. While amino acids and bile acids mainly decreased in concentration compared to placebo, fatty acids and steroids increased. For acyl carnitines, both increases and decreases were observed, potentially depending on the side chain length (short-chain carnitines tend to decrease, medium-chain carnitines tend to increase). In contrast to medium-chain carnitines, short-chain carnitines appeared unaffected by amphetamine treatment 3 h after intake, but showed a dramatic decrease 7.5 h post administration. Typical changes over time for four acyl carnitines and four long-chain fatty acids are exemplarily depicted in Figure 3. Additionally, five metabolites were assigned based on pathway analysis directly from the feature list, but could not be confirmed by MS/MS data. They are indicated in Table S1 as level 3 identifications.

### 2.4. Effects of Mephedrone on the Human Metabolome

**Figure 4:**
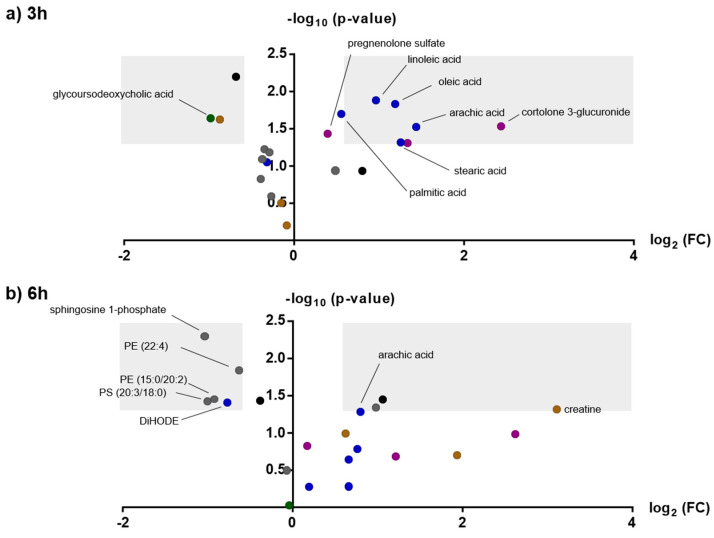
Volcano plots of selected, identified features 3 h (**a**) and 6 h (**b**) after nasal mephedrone consumption. Different compound classes are depicted in different colors: long-chain fatty acids (dark blue), bile acids (green), amino acids (brown), phospholipids (grey). All other features remained black. Compounds in light grey areas can be considered as particularly interesting (−log10 *p*-value > 1.3; log2 foldchange > 0.6 or < −0.6).

In total, 10,664 features were extracted after deconvolution in Progenesis Qi in all four methods. Initial search for features with an infinite fold-change and low abundance at time points 0 (before mephedrone administration) revealed two features: 7.32_177.1134n (*p*-values 0.019 and 0.046 after 3 h and 6 h, respectively) and 4.42_208.0958 *m/z* (*p*-values 0.089 and 0.039 after 3 h and 6 h, respectively). Based on their accurate masses and fragment ions they were identified as mephedrone, and its metabolite 4-carboxymephedrone. Normalized peak areas vs. time after mephedrone intake of mephedrone and its metabolite are given in the Supplementary Information Figure S1. High mephedrone and 4-carboxymephedrone levels were found 3 h after intake, while 6 h later, they remained detectable, albeit at low levels. Further application of the set criteria to the abundance > 500 cps in more than 8 samples reduced the initial number of features to 2137. Univariate analysis (paired t-test, *p* < 0.05; fc > 1.5) finally resulted in 68 features with time-dependent changes after mephedrone consumption, with 21 identified with a confidence level of 2 or lower either at time point 1, time point 2 or both. The respective volcano plots are given in Figure 4. Detailed statistical results and analytical characteristics are summarized in Table S2 of the Supplementary Data. Main compound classes were long-chain fatty acids and steroids, which mainly increased after mephedrone consumption.

### 2.5. Comparative Analysis of MDMA, Amphetamine and Mephedrone Effects

**Figure 5:**
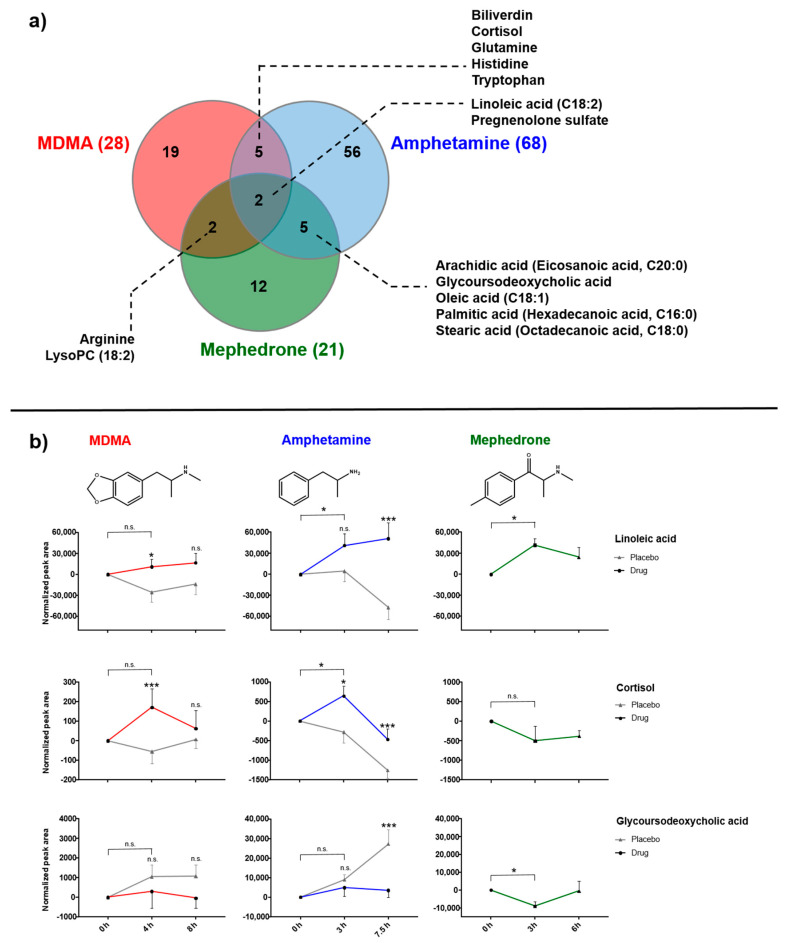
(**a**) Comparative analysis of identified, influenced features following MDMA (red), amphetamine (blue) and mephedrone (green) intake displayed as Venn-diagram. (**b**) Detailed changes over time for linoleic acid (upper panel), cortisol (middle panel) and glycocholic acid (lower panel). Colored lines indicate drug, light grey lines placebo intake, respectively. Data points represent mean and standard error of mean (SEM) of 16 and 13 replicates for MDMA and amphetamine, respectively. Mephedrone data display mean and standard deviation (SD) of *n* = 5 replicates. Statistical comparison was performed between placebo and drug intake and between time points 0 and 1 using paired *t*-tests (*p* < 0.05; n.s. > 0.05; * 0.01–0.05; *** 0.0001–0.001).

To compare the findings from the three individual studies with MDMA, amphetamine, and mephedrone, in a first step individual, identified features were submitted to meta-analysis in Metaboanalyst 4.0. Meta-analysis in Metaboanalyst requires at least 25% features to be common [33], which was higher than the overlap in our dataset. As displayed in the Venn-diagram in Figure 5a, only two compounds—linoleic acid and pregnenolone sulfate—could be identified as significantly changed by all three stimulants. Further five overlaps were observed between MDMA/amphetamine (biliverdin, cortisol, glutamine, histidine, and tryptophan) and amphetamine/mephedrone (arachidic acid, glycoursodeoxycholic acid, oleic acid, palmitic acid, stearic acid). Only arginine and LysoPC (18:2) were altered by both MDMA and mephedrone. As shown in Figure 5b, linoleic acid and cortisol significantly increased after MDMA or amphetamine intake compared to placebo. Mephedrone-induced changes on linoleic acid appeared more closely related to those observed for amphetamine; cortisol was unaffected by mephedrone. MDMA did not have an influence on glycoursodeoxycholic acid concentrations, while amphetamine intake resulted in decreased levels compared to placebo. Mephedrone slightly lowered glycoursodeoxycholic acid concentrations in a time-dependent manner.

**Figure 6:**
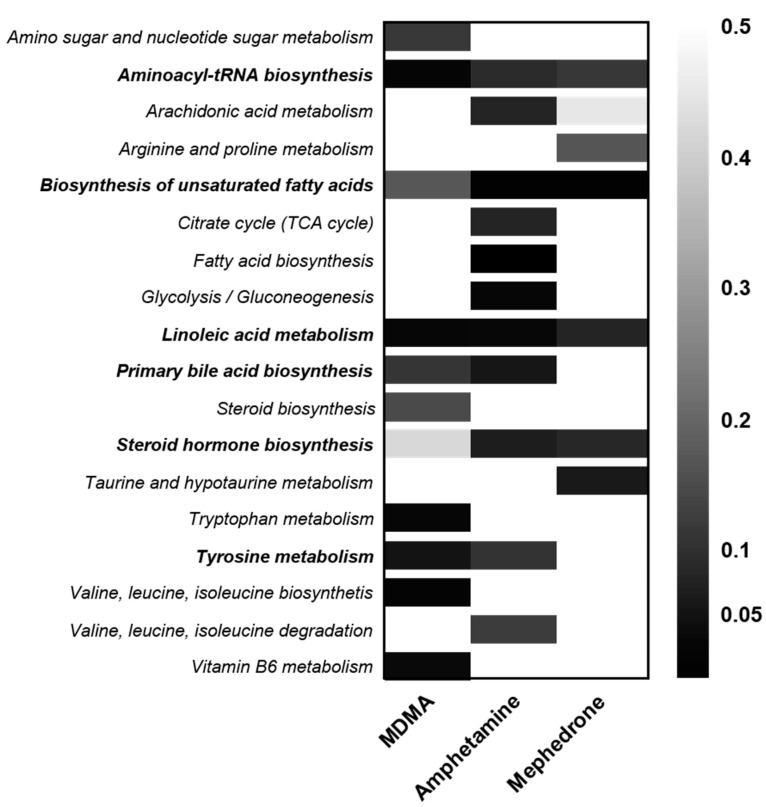
Heat map of the combined (mummichog and gene set enrichment analysis (GSEA)) *p*-value for all pathways identified as significantly altered in a time-dependent manner (before vs. after drug intake) by MDMA, amphetamine and mephedrone. High significance for a pathway in colored black (0), descendent significance is indicated in lighter colors. Pathways significantly affected by at least two drugs are written in bold.

Unambiguous identification is still the bottleneck of untargeted metabolomic profiling. Therefore, the incompleteness of the definitely identified metabolites hinders a robust comparison. Therefore, a second analysis aimed at affected biological pathways or compound classes in general, rather than on individual metabolites. Algorithms such as mummichog or gene set enrichment analysis (GSEA) predict potentially affected biologicals pathways without prior individual metabolite identification. Applying the combination of mummichog and GSEA algorithms to focus on time-dependent (before vs. after drug intake (time point 1, MDMA 4 h, amphetamine 3 h, mephedrone 3 h, respectively)) influences on entire pathways based on the MS data has revealed 9, 14, 5 pathways as significantly affected by MDMA, amphetamine, and mephedrone, respectively. An overview of the combined (mummichog and GSEA) *p*-values for all pathways in relation to the different stimulant drug is given in Figure 6 and indicates certain pathways involved in all three (aminoacyl-tRNA biosynthesis; linoleic acid metabolism) or a combination of two stimulants (biosynthesis of unsaturated fatty acids, primary bile acid biosynthesis, steroid hormone biosynthesis, tyrosine metabolism). Typical compounds of a pathway as well as *p*-values for the different algorithms are provided in Table S3 of the Supplementary Information. Findings from pathway analysis correspond well to those of individually identified compounds (Figure 2 and Figure 4, Tables S1 and S2).

## 3. Discussion

The (human) plasma metabolome is highly dynamic and influenced not only by the use of (prescription) drugs but also by diet, sport, sleep cycles, etc. Many endogenous metabolites also underlie circadian variation. Reliable detection of drug influence on the metabolome, therefore, requires standardized conditions to exclude confounding factors. The clinical studies used for this comparative study were performed under controlled conditions with a known, defined drug dose and purity, supervised drug administration, and regular sample collection at predefined time points. However, they were performed individually and independently and were not initially planned for the herein presented metabolic profiling but for separate pharmacological/pharmacokinetic questions. Based on the different aims and designs of the studies, not all parameters (such as drug dose, administration route, collected time points, number of participants, blood preservatives, placebo-control) were identical. Limitations possible arising from the differing study conditions will be discussed in the following. Nevertheless, as controlled drug administration studies in humans are scarce and often limited due to ethical restrictions in many countries, collected data and samples are highly valuable also for other research questions, such as the presented metabolomics study.

Different doses of the psychostimulants were administered (125 mg MDMA, 40 mg D-amphetamine (referred to as amphetamine), 100 mg mephedrone). MDMA and amphetamine doses can be considered as “equivalent” with regard to cardiovascular effects. Both doses are in the rather high range of recreational drug use known to produce the typical psychostimulant effect, but are still considered as “safe” [5]. The chosen mephedrone dose also lies within the typical range used for recreational purposes and represented a safe dose for the participants. Considering their slightly different pharmacological action, the selected doses can be considered as “equipotent” as they represent typical recreational user doses. Despite the different administration between MDMA/amphetamine (oral ingestion) and mephedrone (nasal insufflation), the drugs were administered using the typical method under recreational conditions. Plasma sampling time differed slightly between studies, but similar time points could be selected for the comparison: time point 0 before drug administration; time point 1 around the expected Tmax (4 h for MDMA, 3 h for amphetamine, and 3 h for mephedrone) and time point 2 in a range of declining plasma concentrations (8 h MDMA, 7.5 h amphetamine, 6 h mephedrone). Sample sizes were different due to initial subject recruiting and availability for reanalysis of the selected time points (16, 13 and 5 for MDMA, amphetamine and mephedrone, respectively). Plasma samples after mephedrone consumption were preserved typically for forensic purposes using sodium fluoride potassium oxalate in contrast to heparin for MDMA and amphetamine samples. It is known that the type of blood used, including different preservatives, can influence the metabolomic profile [34]. However, we first identified metabolites affected by each psychostimulant separately through comparison before and after drug intake or versus placebo. Within each study group, all samples were collected in the same way. While both MDMA and amphetamine were administered in a placebo-controlled, crossover design with minimal discrepancies and within the same laboratory, no placebo was taken during the mephedrone study, which has been initially designed for pharmacokinetic investigations only. Following this, the major issue for reliable comparison was the lack of placebo control for mephedrone samples, allowing comparative analysis on time-dependent changes only. Previous studies on MDMA have, however, revealed time as an additional factor for observed endogenous changes, e.g., of certain amino acids, rather than drug intake [23]. Therefore, changes in metabolites observed after mephedrone intake should not necessarily be considered as drug-related but might rather be due to circadian influences. Nevertheless, some compounds after mephedrone ingestion behaved quite similarly to those after treatment with MDMA or amphetamine (e.g., linoleic acid, Figure 5), while other compounds more closely resembled the placebo course (cortisol, Figure 5). This allows the conclusion that at least metabolites affected by mephedrone and one of the other drugs (against placebo) are likely to be mephedrone-related rather than circadian.

In general, peak areas were smaller during amphetamine and mephedrone sample measurements compared to MDMA data (time difference in data acquisition about two years), but evaluation of SST and pooled plasma samples indicated sufficient instrument performance in similar ranges as expected from the initial method evaluation [31]. As already observed in former SST measurements, also during amphetamine and mephedrone batch analysis, bile acids chenodesoxycholic acid and deoxycholic acid showed decreasing trends. To avoid potential analyte instability in processed samples or instrument performance to confound data interpretation, all study samples were analyzed in randomized order. Combination of high-resolution MS with different chromatographic systems allowed the simultaneous detection of thousands of features. After peak picking, alignment and deconvolution, peaks typically require identification prior to further biological analysis [33]. Measured MS and MS/MS signals need to be annotated to known metabolites. Even with high mass accuracy several metabolites can match to a single MS peak. MS/MS information provides additional identification power, but even with spectral matching to different databases the coverage of metabolites remains low [35]. The incompleteness of identification of significantly changed features should be kept in mind when comparing the effects of different psychostimulants on individual metabolites. An additional approach is the use of algorithms such as mummichog or GSEA [35,36,37,38]. The mummichog algorithm infers pathway activities from a ranked list of MS peaks identified by untargeted metabolomics. It implements an over-representation analysis method to evaluate pathway-level enrichment based on significant features. A list of metabolites is inferred from all *m/z* features by accepting all potential matches including isotopes and adducts. The tentative matches are then mapped onto known metabolic pathways of the human metabolism [35]. The GSEA method extracts biological meaning from a ranked gene list. In contrast to mummichog, it considers the overall ranks of MS peaks without taking significance cutoffs into account [36]. This makes GSEA superior over mummichog to detect subtle and consistent changes. The major limitation of these algorithms is that one single feature might be matched to multiple metabolites from different pathways. The result is an overrepresentation in the number of potentially affected pathways. Still, the application of such algorithms can give hints for affected biological pathways that should then be further investigated in targeted studies.

In the present studies, various metabolites that were identified as influenced by MDMA, amphetamine, and mephedrone derived from energy metabolism in general (compound classes of acyl carnitines, fatty acids, bile acids; affected pathways “biosynthesis of unsaturated fatty acids” and “primary bile acid biosynthesis”), steroid biosynthesis and certain amino acids as will be discussed in the following.

### 3.1. Energy Metabolism

Our findings point to an increased energy demand especially caused by amphetamine, but also by mephedrone and amphetamine. Increases in high numbers of fatty acids, bile acids and some acyl carnitines were observed. In humans, the main source of fat is ingestion and/or storage in form of triglycerides or cholesterol. When needed, lipid metabolism starts either from dietary fats or from stored lipids and involves degradation of triglycerides to fatty acids. Fatty acid catabolism produces energy (adenosine triphosphate, ATP) by mitochondrial beta-oxidation yielding acetyl-CoA, which in turn enters the citrate cycle. Long-chain fatty acids (more than 14 C-atoms) require prior conversion with L-carnitine to acyl-carnitines to cross the mitochondrial membrane [39]. Bile acids—derived from the catabolism of cholesterol—are physiological detergents that facilitate absorption and transport of fats through formation of micelles [40,41].

#### 3.1.1. Fatty Acids

The highest number of increased fatty acids was observed for amphetamine 7.5 h post ingestion, followed by mephedrone (Tables S1 and S2, Figure 2 and Figure 4, reference [24]). MDMA seemed to have only a minor influence on the level of fatty acids in our study, similar to previous untargeted human data that detected no changes in free fatty acids for MDMA [22]. Mephedrone appears to be more closely related to amphetamine than MDMA in terms of fatty acid metabolism. In principle, elevation of free plasma fatty acids may result from degradation of storage fat (e.g., triglycerides) or from new fatty acid biosynthesis. Former studies have shown that amphetamine can affect lipid metabolism by decreasing postprandial increase of plasma triglycerides and elevating free plasma fatty acids in rats [42]. Moreover, lowered plasma triglycerides have been reported in methamphetamine dependent patients [43]. Methamphetamine treatment in rats also pointed to an accelerated lipid metabolism indicated by an initial decrease in glycerol lipids, but rather constant amounts of fatty acids after acute treatment. Following five days of continuous methamphetamine administration, urinary concentrations of palmitic acid and stearic acid were found to decrease [18]. Following this and our data from the three psychostimulant drugs in humans, lipid metabolisms appears to play a crucial role as an energy source after intake of stimulant drugs. The increase in fatty acids occurred with a certain delay (e.g., after 7.5 h rather than 3 h after amphetamine intake). It seems likely that amphetamine and its derivatives activate triglyceride catabolism to form free fatty acids followed by an elevated beta-oxidation once other energy sources (glucose) are spent. In particular, the short-chain fatty acid 3-hydroxybutyrate was altered after amphetamine intake in our study. The compound 3-hydroxybutyrate is the only acetyl-CoA synthesized in the liver, which is used as an alternative energy source by the brain when blood glucose is low. In addition, the long-chain fatty acids palmitic acid, stearic acid, oleic acid, linoleic acid or their derivatives were found to be elevated (Figure 3). Phospholipids present in the cell membranes contain significant proportions of palmitic and stearic acids [44]. Palmitic acid is also known to make up around 30% of human fat storage [45]. In addition to energy metabolism, fatty acids have also other functions. There is evidence that the even-numbered saturated fatty acids, such as palmitic acid, impact inflammation, among others. Linoleic acid, that increased after intake of MDMA, amphetamine and mephedrone, is an essential omega-6 fatty acid, which is used in the biosynthesis of prostaglandins via arachidonic acid. The omega-6 fatty acid arachidonic acid mainly acts as an eicosanoid precursor with eicosanoids associated with inflammatory diseases [44]. For MDMA, effects on inflammation pathways were already discussed previously based on the detection of hydroxyl-eicosatetraenoic acid (5-HETE), dihydroxy-eicosatetraenoic acid (5,12-diHETE) and oxo-octadecanedienoic acid (13-oxoODE) [24]. None of the aforementioned metabolites were detected during amphetamine or mephedrone data evaluation. In contrast to amphetamine and mephedrone, reactive quinone-metabolites of MDMA can produce semiquinone radicals which can cause the formation of reactive oxygen species (ROS), which can lead in turn to systemic oxidative stress and inflammation processes [46,47].

#### 3.1.2. Acyl Carnitines

Particularly after amphetamine intake, decreases in short-chain acyl carnitines 7.5 h after intake and increases in medium- and long-chain acyl carnitines, even 3 h after intake were observed (Figure 3). Following MDMA administration, carnitine and propionylcarnitine were shown to decline [24]. Similar to our results, Nielsen et al. identified elevated levels of medium-chain acyl carnitines (2-octenoylcarnitine, decanoylcarnitine, 9-decenoylcarnitine, dodecanoylcarnitine, tetradecenoylcarnitine) in retrospective human data of MDMA consumers [22]. The longer the fatty acid, the higher the amount of ATP that can be gained through beta-oxidation. Switching of short-chain acyl carnitines (decreased in our study) to medium- or long-chain acyl carnitines (increased in our study) thereby might improve the overall energy yield. Perrine et al. described increased levels of carnitine in heart muscle tissue of rats treated with MDMA and concluded that the increased energy demand caused by MDMA is covered by an increased beta-oxidation of fatty acids [48]. Vanaveski et al. observed increases of medium-chain acyl carnitines after repeated amphetamine administration in 129Sv mice. From the elevation of the ratio between long chain acyl carnitines to carnitine, the authors also proposed an increased participation of acyl carnitines to account for an additional energy demand [49]. It is also known that immune cells such as leukocytes are enriched in carnitines. Again, the influence of carnitines on functions of immune cells predominantly relies on carnitine-dependent energy production from fatty acids which makes them crucial to the maintenance of cell membrane structure and cell viability. Normal carnitine metabolism might counteract an exaggerated production of ROS—e.g., from drug intake—protecting tissues against irreversible damage during the acute inflammatory response [50].

#### 3.1.3. Bile Acids

Particularly following amphetamine ingestion, plasma levels of various bile acids were significantly lowered in our study. After mephedrone treatment, only glycoursodeoxycholic acid levels decreased time-dependently. As already described for fatty acids, following MDMA intake no altered bile acids were detected (Figure 5) [22,24]. The distinction between different bile acids is minute, depends only on presence or absence of hydroxyl groups on positions 3, 7, and 12. Their main function lies in the emulsification of fats facilitating fat excretion, absorption, and transport. Increased levels of free fatty acids observed herein in response to amphetamine and to a lower extent to mephedrone might therefore require higher capacities of bile acids for the transport, which in turn lowers free bile acid concentration in plasma.

### 3.2. Steroid Metabolism

The steroid and steroid hormone biosynthesis pathway appeared to be significantly affected by the stimulant drugs MDMA, amphetamine and mephedrone (Figure 2, Figure 3 and Figure 4 [24]). Psychostimulant drugs are considered chemical stressors in general. Their effects on corticosteroids such as cortisol have been described in several investigations following MDMA or amphetamine consumption [16,51,52,53,54]. Our data indicated similar results for cortisol and pregnenolone-sulfate. Other steroids could not be identified with sufficient confidence. Untargeted metabolomics often lacks sensitivity compared to specific targeted methods. MS/MS spectra—the prerequisite for unambiguous identification—are often only obtained for features with sufficiently high abundance. Particularly steroids, being present at low concentrations in the blood, might require higher sensitivity [31]. Cortisol is a glucocorticoid steroid, which is released in response to stress or low blood-glucose concentration and it is also a marker for serotonergic activity [55]. Cortisol levels were significantly elevated after 4 h and 3 h post MDMA and amphetamine intake, respectively. Similar trends were not observed in the investigated five mephedrone subjects (Figure 2). In contrast to our findings, Olesti et al. observed significant effects on cortisol also after mephedrone consumption in rats [16]. However, our study design lacks the placebo control, which hampers a definite conclusion. Pregnenolone sulfate is a neuroactive steroid synthesized from pregnenolone [56]. Pregnenolone formation from cholesterol is the initial step of steroid-synthesis. Pregnenolone is further metabolized to pregnenolone sulfate and progesterone. Progesterone reacts with 17-hydroxyprogesterone—the starting point for further glucocorticoid synthesis, e.g., cortisol. Pregnenolone sulfate itself regulates many brain functions by binding to γ-aminobutyric acid (GABA) and N-methyl-D-aspartate (NMDA) type receptors with high affinity [57]. It has also been associated with generalized anxiety disorders or with generalized social phobia compared to healthy male subjects [58,59].

## 4. Materials and Methods

### 4.1. Clinical Studies

#### 4.1.1. MDMA Study

Study details have been published elsewhere [23,24,30]. Briefly, 16 healthy participants (eight male, eight female, age 24.4 ± 2.2 years, range 20−27, body mass index of 22.76 ± 2.1 kg/m^2^, heavy smokers with >10 cigarettes/day and illicit drug use excluded, standardized lunch served at session days) underwent four different treatment conditions (double blind, placebo-controlled, crossover design), from which only placebo and MDMA session were considered for comparison in the present study. Washout periods between session days were at least 10 days. On the session days, MDMA (125 mg p.o., corresponding to 1.8 ± 0.2 mg/kg body weight) or placebo was ingested at 10 a.m. Plasma samples (lithium heparin) used in this study were collected at 8 a.m. (=time point 0; baseline), 2 p.m. (=time point 1; 4 h post-dose), and 6 p.m. (=time point 2; 8 h post-dose). Only already measured and pre-evaluated data published recently were included in the present study for comparison reasons [24]. The clinical study was conducted at the University Hospital of Basel. The study was approved by the Ethics Committee of the Canton of Basel, Switzerland and registered at ClinicalTrials.gov (NCT01771874). All subjects provided written informed consent and were paid for their participation. All samples were stored at the Zurich Institute of Forensic Medicine at −80 °C and were never thawed prior to analysis.

#### 4.1.2. Amphetamine Study

Samples from a double-blind, placebo-controlled, cross-over design with four experimental test sessions conducted at the University Hospital in Basel, Switzerland as described elsewhere [5] were used in the current study. Out of the four sessions, only plasma samples (lithium heparin) collected during placebo and D-amphetamine (in the manuscript referred to as amphetamine; 40 mg p.o.; administered at 9 a.m.) sessions at baseline (prior to placebo or amphetamine administration, time point 0), 3 h post-dose (time point 1) and 7.5 h (time point 2) were analyzed as described below. Complete set of samples was available for 13 participants (6 females, 7 males; 28 ± 5 years old [mean ± SD]; range, 25–45 years; body weight, 71.5 ± 11.4 kg, heavy smokers with >10 cigarettes/day and illicit drug use excluded, standardized lunch served at session days). The washout period between sessions was at least 10 days. The study was conducted in accordance with the Declaration of Helsinki and approved by the Ethics Committee northwest Switzerland (EKNZ). All of the participants provided written informed consent before participating in the study, and they were paid for their participation. The study was registered at ClinicalTrials.gov (NCT03019822). All samples were stored at the Zurich Institute of Forensic Medicine at −80 °C and were never thawed prior to analysis.

#### 4.1.3. Mephedrone Study

The study was conducted by King’s College London; details are given elsewhere [60]. Mephedrone powder self-administration (100 mg, corresponding to 1.4 ± 0.2 mg/kg body weight; ingested at 9 a.m.) was carried out via nasal insufflation using a straw under medical supervision. In contrast to the MDMA and amphetamine studies no placebo group was included. From all available plasma samples (sodium fluoride potassium oxalate (NaF/KOx)), baseline (time point 0), 3 h post mephedrone administration (time point 1), and 6 h post mephedrone administration (time point 2) samples were included into the current study given a complete set of samples for 5 (out of 6) participants (healthy non-smoking men between 18 and 40 years who were occasional users of mephedrone or other stimulant drugs, body weight 70.5 ± 12.1 kg, standardized lunch served at session day). All participants were compensated with London living wage for the time they have been participating in the study. Ethical approval was obtained from the Riverside National Research Ethics Service (16/LO/1342). All samples were stored at the Zurich Institute of Forensic Medicine at −80 °C and were never thawed prior to analysis.

### 4.2. Chemicals and Reagents

The substances 1-Methylhistidine, adenine, adenosine, arginine, azelaic acid, chenodeoxycholic acid, cholic acid, citrulline, cortisol, cortisone, creatinine, deoxycholic acid, glutaric acid, glycocholic acid, hippuric acid, inosine, isoleucine, leucine, l-pyroglutamic acid, methionine, methylmalonic acid, mevalonolactone, N,N-dimethylglycine, nicotinic acid, p-aminobenzoic acid, phenylalanine, proline, raffinose, riboflavin, taurine, taurocholic acid, tryptophan, and uracil were purchased from Sigma-Aldrich (Buchs, Switzerland). Deuterated and heavy-labeled internal standards (IS) adenosine ribose-D1, arginine-13C6, caffeine 3-methyl-13C, carnitine trimethyl-D9, creatinine N-methyl-D3, deoxycholic acid-D4, D-fructose 13C, glycine-13C2, glycocholic acid-D4, hippuric acid 15N, kynurenine-D4, leucine-D10, lysine-D4, phenylalanine-D1, proline 15N, serine-D3, tryptophan-D5 and uric acid-15N2 were purchased from Cambridge Isotope Laboratories (Cambridge, MA, USA), and were delivered by ReseaChem Life Science (Burgdorf, Switzerland) or Sigma-Aldrich (Buchs, Switzerland). Water, acetonitrile (ACN), methanol (MeOH) of HPLC grade were obtained from Fluka (Buchs, Switzerland). All other chemicals used were from Merck (Zug, Switzerland) and were of the highest grade available.

### 4.3. Sample Preparation

Protein precipitation was performed from 150 µL of plasma mixed with 30 µL of the IS mixture (given in detail in references [24,31]) by addition of 450 µL ice-cold MeOH/acetone (9:1 *v*/*v*). After vortex mixing and centrifugation (10 min, 14,000× *g*), 250 µL of the supernatant was transferred into two filter vials (0.45 µm PTFE, Thomson Instrument company, California, USA). Samples were stored at −20 °C until analysis on liquid chromatography–high resolution mass spectrometry (LC-HRMS) as described below. Additionally, pooled samples for each study with 10 µL from every sample were prepared in the same manner.

### 4.4. LC-HRMS Analysis

All samples were subjected to MS measurements in randomized order using a Thermo Fischer Ultimate 3000 UHPLC system (Thermo Fischer Scientific, San Jose, CA, USA) coupled to a high-resolution TOF instrument (TripleTOF 6600, Sciex, Concord, ON, Canada). Mobile phases A and B consisted of 10 mM ammonium formate with 0.1% (*v*/*v*) formic acid in water and 0.1% (*v*/*v*) formic acid (FA) in MeOH, respectively. Mobile phases C and D were 25 mM ammonium acetate and 0.1% (*v*/*v*) acetic acid in water and 0.1% (*v*/*v*) acetic acid in ACN, respectively. Methodological details and performance evaluation have been published before [31]. Briefly, two different columns—RP and HILIC—were used for chromatographic separation. A Waters (Baden-Daettwil, Switzerland) XSelect HSST RP-C18 column (150 mm × 2.1 mm, 2.5 µm particle size) was applied as RP phase with gradient elution using mobiles phase A and B, a flow rate of 0.5 mL/min (0.7 mL/min after 15 min) and a run time of 20 min. HILIC separation was achieved on a Merck SeQuant ZIC HILIC column (150 mm × 2.1 mm, 3.5 µm particle size) using gradient elution with mobiles phases C and D and a flow rate of 0.5 mL/min over 15 min. The column oven was set to 40 °C and injection volume was 1 µL for all samples.

HRMS and MS/MS data were acquired using TOF-MS only and information dependent data acquisition (IDA), both in positive and negative ionization mode. Analysis was performed with a DuoSpray ion source at a resolving power (full width at half-maximum at *m/z* 400) of 30,000 in MS and 30,000 in MS2 (high-resolution mode) or 15,000 (high-sensitivity mode) in positive ionization mode. Automatic calibration was obtained after every fifth sample using atmospheric-pressure chemical ionization (APCI) positive calibration solution (Sciex) in the positive ionization mode and after every third sample using APCI negative calibration solution (Sciex) in the negative ionization mode. The TOF-MS method was composed of a TOF-MS scan over a mass range of *m/z* 50 to *m/z* 1000 (accumulation time 100 msec, CE 5 eV). Additionally, about 20% of the samples were measured in IDA scan mode. The IDA method consisted of a TOF-MS scan over a mass range of *m/z* 50 to *m/z* 1000 (accumulation time 50 msec, CE 5 eV). IDA experiments (accumulation time for each IDA experiment of 100 msec, CE 35 eV with a CE spread of 15 eV) were performed after dynamic background subtraction on the four most intense ions with an intensity threshold above 100 cps and exclusion time of 5 s (half peak width) after two occurrences in high sensitivity mode.

### 4.5. Quality Control

An SST mix given in detail in references [24,31] and a pooled sample for each data set (amphetamine or mephedrone, respectively) was measured after every fifth sample for QC reasons checking data reproducibility by retention time and peak area. All peaks of the SST mix were manually integrated using Multiquant version 3.0.4 (Sciex, Concord, ON, Canada); QC samples were evaluated for their %CV in Progenesis Qi version 2.4 (Nonlinear Dynamics, Waters Corp, Milford, MA, USA). %CVs lower than 30% were considered acceptable for following data interpretation.

### 4.6. Data Pre-Processing and Identification

Progenesis QI was used for data-preprocessing, alignment, deconvolution, peak picking, initial data normalization and filtering of TOF data only. Data files of the IDA scan mode were incorporated in the software for identification purposes only. Every run in the batch was automatically compared for similarity to all other runs and the one with highest similarity to all other runs was picked as a reference run for data alignment and normalization. Peak picking parameters were as follows: automatic sensitivity method, sensitivity value 3, no minimum peak width and no retention time limits, ion species [M+H]^+^, [M+2H]^2+^, [M+H-H_2_O]^+^, [M+NH_4_]^+^, [M+Na]^+^, [M+2Na]^2+^, [M-H]^−^, [M-2H]^2−^, [M-H_2_O-H]^−^, [M+Na-2H]^−^, [M+FA-H]^−^. Features were automatically deconvoluted based on the same retention time and ion masses that differ by an amount equal to the mass difference between two experimental adducts. For normalization, the method in Progensis QI previously evaluated as best suited for MDMA data [24] was applied to amphetamine and mephedrone data sets. Thereby, a factor was calculated that was multiplied by all ion abundances for all compounds for each sample to allow recalibration to the selected reference run. A quantitative abundance ratio of all detected ions was calculated between the normalized run and the reference run sample (pooled sample 15, 6, 9, and 7 for RP pos, HILIC pos, RP neg, and HILIC neg, respectively for amphetamine batches; pooled samples 6, 2, 8, and 1 for RP pos, HILIC pos, RP neg, and HILIC neg, respectively for mephedrone batches) Mean %CVs of all features were reduced by 5% for amphetamine data, while no difference was observed for mephedrone data.

Identification was performed by searching against an in-house library and against different online databases including METLIN [61], the Human Metabolome Database [62] (HMDB, V4.0), NIST 2014 [63] and Lipidblast [64]. Final identification results were classified on the different levels of identification confidence suggested by the metabolomics standard initiative (MSI) [32]: confirmation using MS/MS information and co-elution with authentic standards (level 1); confirmation through comparison of experimental MS/MS spectra with online databases (level 2); and annotation to putatively characterized compound classes (level 3).

### 4.7. Statistical Evaluation

#### 4.7.1. Amphetamine

Data tables exported from Progenesis Qi where filtered by selecting features with an abundance > 500 (counts per seconds, cps) in at least 10 samples. Different statistical methods were applied to filter for potentially significantly changed compounds. Paired t-tests (*p* < 0.05, two-tailed) were performed between amphetamine treatment and placebo at each time-point (3 h and 7.5 h post-dose). Additionally, only data with overall fold-changes exceeding 1.5 were selected. To account for the multilevel nature of the data set, mixed effect model calculations taking into account a random effect for the crossover design of the study were performed in R Studio (version 1.2.5033). To consider inter-day variability, the baseline level (each feature intensity at t0 after log transformation) was added as an explanatory variable into the model that described the target size (log feature intensity at t1 or t2) through the model terms treatment (placebo or amphetamine), week (1–4 in randomized order), interaction between treatment and week, log feature intensity at baseline (t0) and a random effect for each subject with the following equation (1):

log(Int_t4.5)ijkl = β0 + β1treatmenti + β2weekj + β3(treatment:week)ij + β4log (Int_t0) + γ + ϵijkl,(1)

Thereby β0 represents the y-axis intercept, β1 describes the difference by treatment (placebo vs. amphetamine), β2 describes the difference in administration week (first placebo second amphetamine or vice versa), β3 is the coefficient of interaction between administration and administration week, β4 describes the correlation between the intensities prior and post treatment, and γ represents a random effect to account for the crossover design of the study. Building an automatic loop, the described test is executed on all features delivering *p*-values with and without Benjamini-Hochberg correction (false discovery rate, FDR) for multiple testing. *p*-values ≤ 0.05 were considered to show a significant treatment effect caused by amphetamine. Raw *p*-values independent of the FDR adjustment where further considered for biological interpretation. The complete R-script is given in the Supplementary Information.

#### 4.7.2. Mephedrone

Data tables exported from Progenesis Qi where filtered including only features with an abundance > 500 cps in at least 8 samples. Paired *t*-tests (*p* < 0.05, two-tailed) were performed between time-points (3 h and 6 h post-dose) vs. baseline.

### 4.8. Comparative Analysis of MDMA, Amphetamine, and Mephedrone

#### 4.8.1. Comparison of Identified Compounds

Significantly changed, identified compounds from the previously published MDMA study [24] were compared by Meta-analysis in Metaboanalyst 4.0 (www.metaboanalyst.ca/) [33,65] and InteractiVenn (http://www.interactivenn.net/) [66] to newly identified features affected by amphetamine and/or mephedrone treatment.

#### 4.8.2. Pathway Analysis

Metaboanalyst 4.0 [33,65] was used for MS peaks to pathway analysis of MDMA, amphetamine and mephedrone focusing on changes between baseline and time points 1 (4 h for MDMA, 3 h for amphetamine and mephedrone, respectively). Previous MDMA data were also filtered to include only features with an abundance > 500 cps in more than 10 samples in accordance with amphetamine and mephedrone statistical analysis. We applied the combined mummichog and GSEA algorithm and the Kyoto Encyclopedia of Genes and Genomes (KEGG) Homo sapiens (human) pathway library. For the mummichog algorithms the default *p*-value cutoff was selected focusing on the top 10% or top 500 peaks, while GSEA represents a cutoff-free method, considering the overall ranks of all features involved. It can therefore detect subtle and consistent changes that might be missed using the mummichog method [33]. Significant pathways (*p* < 0.05) for each compound either in mummichog or in GSEA were further evaluated applying univariate statistics as described above and MS/MS information if available.

## 5. Conclusions

Changes of the metabolome were studied for the first time in an untargeted way after controlled administration of the psychostimulants amphetamine and mephedrone to humans and results were compared to a former study with MDMA. The intake of stimulants resulted in an elevated energy demand, especially for amphetamine, followed by mephedrone and far less pronounced for MDMA. Overall, mephedrone effects appeared to be more closely related to amphetamine than to MDMA, which parallels with its known pharmacological actions. For more detailed comparative analysis and correlation to drug actions, quantitative results for a higher number of metabolites from the now identified compound classes and pathways will be necessary. Our presented data can provide the basis for such in-depth targeted metabolome studies in order to evaluate and correlate pharmacological action and search for potential (indirect) biomarkers of drug use.

## Supplementary Materials

The following data are available online at https://www.mdpi.com/2218-1989/10/8/306/s1, R-Script for calculation of *p*-values using mixed-effect model statistics, Figure S1: Normalized peak areas for the features identified as mephedrone and 4-carboxymephedrone 3 h and 6 h after mephedrone administration to five human subjects, Table S1: Identified metabolites that showed significant changes between amphetamine and placebo intake, sorted according to compound classes, Table S2: Identified metabolites that showed significant changes before and after mephedrone intake, sorted according to compound classes, Table S3: Significantly affected pathways returned from MS peaks to pathway analysis in Metaboanalyst 4.0. Given are main pathway compounds and *p*-values for the applied algorithms mummichog, GSEA and the combination of both for psychostimulant. *p*-values < 0.1 are indicated in bold print. n.d. means not detected.
