# Supplementary material for: Comparative Untargeted Metabolomics Analysis of the Psychostimulants 3,4-Methylenedioxy-Methamphetamine (MDMA), Amphetamine, and the Novel Psychoactive Substance Mephedrone after Controlled Drug Administration to Humans
Source: Metabolites. 2020 Jul 27;10(8):306. doi: 10.3390/metabo10080306 (PMC7465486; doi:10.3390/metabo10080306)
Supplement: Supplementary file 1 [file metabolites-10-00306-s001.pdf]

Supplementary information

Comparative untargeted metabolomics analysis of the psychostimulants 3,4-methylenedioxymethamphetamine (MDMA), amphetamine, and the novel psychoactive substance mephedrone after controlled drug administration to humans

Andrea E. Steuer[1], Daria Kaelin[1], Martina I. Boxler[1], Lisa Eisenbeiss[1], Friederike Holze[2], Patrick Vizeli[2], Joanna Czerwinska[3], Paul I. Dargan[4,5], Vincenzo Abbate[3], Matthias E. Liechti[2], Thomas Kraemer[1]

[1] *Department of Forensic Pharmacology & Toxicology, Zurich Institute of Forensic Medicine, University of Zurich, Switzerland*

[2] *Department of Biomedicine and Department of Clinical Research, Division of Clinical Pharmacology and Toxicology, University Hospital Basel, University of Basel, Basel 4056, Switzerland*

[3] *King's Forensics, Department of Analytical, Environmental and Forensic Sciences, King's College London, London, UK.*

[4] *Clinical Toxicology, Guy's and St Thomas' NHS Foundation NHS Trust, London, UK*

[5] *Clinical Toxicology, Faculty of Life Sciences and Medicine, King's College London, London, UK*

Corresponding author:

PD Dr. Andrea E. Steuer

University of Zurich/ Zurich Institute of Forensic Medicine (ZIFM)

Department of Forensic Pharmacology & Toxicology

Winterthurerstrasse 190/52

CH-8057 Zurich, Switzerland

Tel.: 0041 446355679; fax: 0041 446356852

E-mail address: andrea.steuer@irm.uzh.ch

OrcID 0000-0001-8983-235

Table S1: Identified metabolites that showed significant changes between amphetamine and placebo intake, sorted according to compound classes. *m/z* values are given for the highest prevalent ion species. Compounds indexed with $^{+}$ under chromatography/ionization were detected in more than one analytical method. Statistical comparison was performed by paired t-tests (< 0.05) and mixed-effect model calculations in R studio: not significant (n.s.) >0.05; *0.01-0.05; ** 0.001-0.01; *** 0.0001-0.01; **** <0.0001. Identification confidence was assigned based on the Metabolomics Standard Initiative (MSI) as follows: confirmation using MS/MS information and co-elution with authentic standards (level 1); confirmation through comparison of experimental MS/MS spectra with online databases (level 2); and annotation to putatively characterized compound classes (level 3).

Compound name	Identification confidence	Compound class	m/z	Chromatography/ ionization	Adducts	p (t1 vs t0)	p (t1,AM vs. P)	p (t1, mixed effect model)	Foldchange t1	p (t2, AM vs. P)	p (t2, mixed effect model)	Foldchange t2
Pentadecanoylglycine	2	acyl amine	317.2788	HSST pos	M+NH4	n.s.	***	**	-1.2	**		-1.2
Propionylcarnitine (C3)	1	acyl carnitine	218.1376	HILIC pos	M+H	**	n.s.		-2.0	**		-2.0
Butyrylcarnitine (C4)	1	acyl carnitine	232.1527	HILIC pos	M+H	*	n.s.		-1.0	*		-1.9
Valerylcarnitine (C5)	2	acyl carnitine	246.1687	HILIC pos	M+H	*	n.s.		-1.1	**		-2.2
Decanoylcarnitine (C10)	1	acyl carnitine	316.2464	HILIC pos	M+H	n.s.	*		2.2	*		4.6
Tetradecenoylcarnitine (C14:1)	1	acyl carnitine	370.2946	HILIC pos	M+H	n.s.	n.s.		1.6	**		2.5
Palmitoylcarnitine (C16)	1	acyl carnitine	400.3417	HILIC pos	M+H, M+Na	*	n.s.	*	1.9	n.s.		4.5
Hydroxyhexadecanoylcarnitine (C16-OH)	2	acyl carnitine	452.2784	HILIC neg	M+K-2H	n.s.	n.s.	*	1.6	n.s.		1.5
Oleoylcarnitine (C18:1)	2	acyl carnitine	426.3567	HILIC pos	M+H	n.s.	*		3.0	n.s.		3.7

Glutamine	3	amino acid	145.0621	HSST neg	M-H2O-H, M-H	*	n.s.		-1.3	n.s.		-1.2
Histidine	1	amino acid	154.0616	HILIC neg	M-H	*	*	*	-1.2	**	**	-1.2
Isoleucine	1	amino acid	130.0869	HILIC neg+	M-H2O-H	n.s.	**		-1.3	n.s.		-1.1
Tryptophan	1	amino acid	239.0587	HILIC neg	M+Cl	n.s.	n.s.		1.1	**	***	-1.4
12,14-Heptacosanedione	2	beta-diketone	431.3824	HSST pos	M+Na	n.s.	n.s.		1.0	*	***	-1.1
Glycocholic acid	1	bile acid	464.3033	HILIC neg	M-H	n.s.	n.s.		-1.1	**		-2.2
Glycoursodeoxycholic acid	2	bile acid	448.3054	HILIC neg+	M-H	n.s.	n.s.		-1.2	***		-2.8
Glycoursodeoxycholic acid 3-glucuronide	2	bile acid	624.3386	HILIC neg	M-H	n.s.	*		-1.3	**		-2.5
Lithocholic acid	2	bile acid	394.3324	HSST pos	M+NH4	n.s.	n.s.		1.0	*	*	-1.2
Sulfolithocholylglycine	2	bile acid	512.2673	HILIC neg	M-2H, M-H, M+Na-2H	*	*		-1.3	n.s.		-1.4
Taurocholic acid	1	bile acid	514.2845	HILIC neg	M-H	n.s.	n.s.		1.2	*		-1.8
Taurodeoxycholic acid	2	bile acid	464.2826	HSST pos	M+H-2H2O	n.s.	n.s.		1.0	**	****	-1.4
Taurolithocholic acid 3-sulfate	2	bile acid	280.6216	HILIC neg	M-2H, M-H, M+Na-2H	*	n.s.	***	-1.1	n.s.		1.0
Bilirubin	2	bilirubin	585.2691	HSST pos	M+H, M+Na +	n.s.	**	***	-2.4	**	*	3.3
Biliverdin	2	bilirubin	583.2532	HILIC pos	M+H	n.s.	***		51	n.s.		9.5
DG(18:0/0:0/20:4n3)	3	diacylglycerol	627.5334	HILIC pos	M+H-H2O	n.s.	n.s.		79.5	*		2.1
Glycylproline	2	dipeptide	171.0769	HILIC neg	M-H	n.s.	n.s.		1.2	*	*	
5-Hydroxyindoleacetic acid	1	indole-3-acetic acid	209.0907	HILIC pos	M+H, M+NH4	n.s.	n.s.		14.7	*		-1.4
Cyclohexaneundecanoic acid	2	long-chain fatty acid	313.2382	HSST neg	M-H, M+FA-H	**	n.s.		1.1	**	***	1.5
Myristic acid (Tetradecanoic acid, C14)	3	long-chain fatty acid	257.1753	HILIC neg+	M-H	***	n.a.		1.2	***		2.1
Myristoleic acid (C14:1)	2	long-chain fatty acid	249.1826	HSST pos	M+Na	n.s.	*	**	-1.1	*	**	-1.1

Palmitic acid (Hexadecanoic acid, C16:0)	1	long-chain fatty acid	255.2324	HSST neg	M-H, M+FA-H, 2M-H	n.s.	n.s.		2.0	**		2.4
Keto-palmitic acid (Oxohexadecanoic acid)	2	long-chain fatty acid	269.2116	HSST neg	M-H	**	**	*	1.3	n.s.		1.1
Hydroxypalmitic acid (HO-hexadecanoic acid)	2	long-chain fatty acid	271.2261	HILIC neg	M-H	n.s.	n.s.		1.7	***		1.5
Palmitoleic acid (C16:1)	2	long-chain fatty acid	253.2167	HILIC neg	M-H	n.s.	n.s.		1.1	****		2.8
Thapsic acid (Hexadecanedioic acid)	1	long-chain fatty acid	285.2068	HILIC neg	M-H	**	n.s.		1.5	**		2.0
Stearic acid (Octadecanoic acid, C18:0)	1	long-chain fatty acid	283.2633	HILIC neg	M-H, M+Na-2H, M+FA-H	**	n.s.		1.1	**	***	1.4
Ketostearic acid (Oxooctadecanoic acid)	2	long-chain fatty acid	297.2425	HILIC neg	M-H, M+Cl	*	n.s.		2.9	*		2.4
Hydroxystearic acid (HO-Octadecanoic acid)	2	long-chain fatty acid	299.2591	HILIC neg	M-H	*	n.s.		1.1	*	**	1.2
Oleic acid (C18:1)	1	long-chain fatty acid	281.2484	HILIC neg	M-H, M+Na-2H, M+FA-H	*	n.s.		1.2	***		2.0
Linoleic acid (C18:2)	2	Long-chain fatty acid	279.2328	HILIC neg+	M-H	*	n.s.		1.3	***		2.0
DiHOME (Dihydroxyoctadecenoic acid)	2	long-chain fatty acid	315.2524	HSST pos+	M+H	n.s.	**	**	-1.1	n.s.		1.0
Arachidic acid (Eicosanoic acid, C20:0)	3	eicosanoic acid	311.2949	HILIC neg	M-H2O-H, M+Na-2H,	**	**		41.0	*		1.6
Eicosenoic acid (C20)	2	long-chain fatty acid	309.2800	HILIC neg	M-H, M+Na-2H	*	n.s.		3.9	***		3.3
Eicosatetraenoic acid (C20:1)	2	long-chain fatty acid	303.2322	HSST neg	M-H	n.s.	n.s.		1.4	**		2.4
13-HDoHE	2	long-chain fatty acid	332.2956	HSST pos	M+NH4	n.s.	n.s.		1.0	*	*	1.1
Caprylic acid (Octanoic acid, C8:0)	3	medium-chain fatty acid	143.1082	HILIC neg		***	n.s.		1.2	n.s.		-1.1
Hydroxycapric acid	2	medium-chain fatty acid	187.1338	HILIC neg	M-H	**	*		2.0	*		2.8
Lauric acid (dodecanoic acid, C12:0)	3	medium-chain fatty acid	199.1703	HILIC neg+	M+Na-2H, M+FA-H	***	n.s.		1.4	n.s.		2.1
Hydroxydodecanoic acid	2	medium-chain fatty acid	215.1658	HILIC neg	M-H	n.s.	n.s.		1.4	***		11.1
Hydroxybutanoic acid	2	short-chain hydroxy acid	103.0397	HILIC neg	M-H	**	n.s.		1.6	*		2.5
Catechol sulfate	2	phenylsulfates	188.9873	HILIC neg	M-H	*	*		3.8	**		-2.0

PC(15:0/18:2(9Z,12Z))	2	phosphocholine	742.5390	HILIC neg	M-H	n.s.	**		1.0	**		-1.1
LysoPC(17:0)	2	phosphocholine	508.3387	HILIC neg	M-H	n.s.	**	***	1.4	*	***	1.1
LysoPE(0:0/16:0)	2	phosphoethanolamine	452.2773	HILIC neg	M-H, M+Na-2H	n.s.	n.s.		1.0	*	*	-1.1
PE(18:1(9Z)/0:0)	2	phosphoethanolamine	480.3066	HILIC pos	M+H	*	n.s.		1.1	*		-1.6
PE(16:0/20:4(8Z,11Z,14Z,17Z))	3	phosphoethanolamine	740.5264	HSST pos	M+H	n.s.	n.s.		1.1	*	*	-1.3
PE(18:2(9Z,12Z)/16:0)	2	phosphoethanolamine	714.5090	HILIC neg	M-H	n.s.	*		-1.2	n.s.		-1.3
Prostaglandin H2	2	prostaglandine	397.2220	HILIC neg	M+FA-H	n.s.	n.s.		1.1	*		-1.1
Cortisol	1	steroid	363.2154	HILIC pos	M+H	*	*		2.1	**		2.9
Cholesterol sulfate	2	steroid	465.3032	HSST neg	M-H	n.s.	n.s.	*	1.3	n.s.		1.0
Pregnenolone sulfate	2	steroid	397.2041	HSST neg+	M-H	n.s.	*		1.5	n.s.		1.3
Tetrahydrodeoxycorticosterone	2	steroid	357.2378	HSST pos	M+Na, M+CH3OH+H	n.s.	n.s.	*	-1.2	n.s.		1.1

Table S2: Identified metabolites that showed significant changes before and after mephedrone intake, sorted according to compound classes. *m/z* values are given for the highest prevalent ion species. Compounds indexed with ⁺ under chromatography/ionization were detected in more than analytical methods. Statistical comparison was performed by paired t-tests (< 0.05): not significant (n.s.) >0.05; *0.01-0.05; ** 0.001-0.01; *** 0.0001-0.01; **** <0.0001. Identification confidence was assigned based on the Metabolomics Standard Initiative (MSI) as follows: confirmation using MS/MS information and co-elution with authentic standards (level 1); confirmation through comparison of experimental MS/MS spectra with online databases (level 2); and annotation to putatively characterized compound classes (level 3).

Compound name	Identification confidence	Compound class	m/z	Chromatography/ ionization	Adducts	p (t1 vs t0)	Foldchange t1	p (t2 vs t0)	Foldchange t2
3-Methylhistidine	2	amino acid	170.0921	HILIC pos	M+H	*	-1.8	n.s.	3.8
Arginine	1	amino acid	175.1190	HILIC pos+	M+H	n.s.	-1.1	*	1.5
Creatine	2	amino acid	132.0761	HILIC pos	M+H	n.s.	-1.1	*	8.6
Glycoursodeoxycholic acid	2	bile acid	448.3061	HILIC neg	M-H	*	-2.0	n.s.	1.0
Palmitic acid (Hexadecanoic acid, C16:0)	1	long-chain fatty acid	255.2326	HILIC neg	M-H	*	1.5	n.s.	1.1
Stearic acid (Octadecanoic acid, C18:0)	1	long-chain fatty acid	283.2633	HSST neg	M-H	*	2.4	n.s.	1.6
Oleic acid (C18:1)	1	long-chain fatty acid	281.2484	HILIC neg	M-H, M+Na-2H, M+FA-H	*	2.3	n.s.	1.6
Linoleic acid (C18:2)	1	long-chain fatty acid	279.2326	HILIC neg	M-H	*	1.9	n.s.	1.7
DiHODE (dihydroxyoctadecadienoic acid)	2	long-chain fatty acid	311.2221	HILIC neg	M-H, M+Na-2H	n.s.	-1.2	*	-1.7

Arachidic acid (Eicosanoic acid, C20:0)	2	long-chain fatty acid	311.2953	HILIC neg	M-H	*	2.7	*	1.7
LPA(18:2(9Z,12Z)/0:0)	2	lysophosphatidic acid	433.2363	HILIC neg	M-H	**	-1.6	*	-1.3
LysoPC(18:2)	2	phosphocholine	520.3373	HSST pos	M+H, M+Na	*	-1.3	n.s.	1.0
PE(15:0/20:2(11Z,14Z))	2	phosphoethanolamine	774.5283	HILIC neg	M+FA-H	n.s.	-1.3	*	-1.9
PE(22:4(7Z,10Z,13Z,16Z)/P-18:0)	2	phosphoethanolamine	800.5595	HILIC neg	M+Na-2H	n.s.	-1.2	*	-1.6
PI(20:4(8Z,11Z,14Z,17Z)/16:0)	2	phosphoinositol	857.5204	HILIC neg	M-H	n.s.	1.4	*	2.0
PS(20:3(8Z,11Z,14Z)/18:0)	2	phosphoserine	858.5785	HILIC neg	M-H, M+FA-H	n.s.	-1.3	*	-2.0
Sphingosine 1-phosphate	2	phosphosphingolipids	378.2412	HILIC neg	M-H	n.s.	-1.2	**	-2.0
Uric acid	2	purine	167.0213	HSST neg	M-H	n.s.	1.7	*	2.1
Cortolone-3-glucuronide	2	steroid	541.2692	HILIC neg	M-H	*	5.4	n.s.	6.2
Pregnenolone sulfate	2	steroid	397.2041	HILIC neg	M-H	*	1.3	n.s.	1.1
Tetrahydroaldosterone-3-gluc	2	steroid	539.2535	HILIC neg	M-H	*	2.5	n.s.	2.3

Fig. S1: Normalized peak areas for the features identified as mephedrone and 4-carboxymephedrone 3 h and 6 h after mephedrone administration to five human subjects.

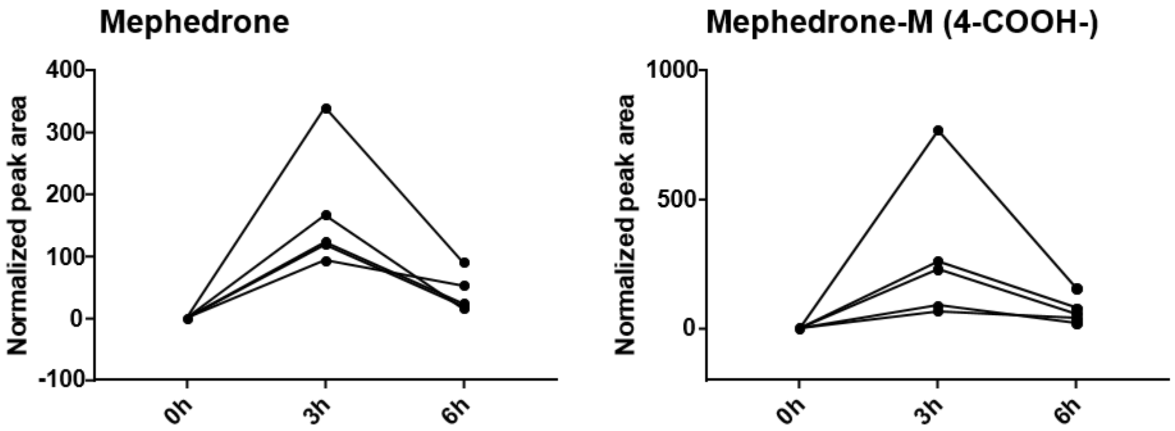

Table S3: Significantly affected pathways returned from MS peaks to pathway analysis in Metaboanalyst 4.0. Given are main pathway compounds and p-values for the applied algorithms mummichog, GSEA and the combination of both for psychostimulant. p-values < 0.1 are indicated in bold print. n.d. means not detected.

**Pathway**	**Main compounds**	**Psychostimulant**	P-values		
			mummichog	GSEA	combined
**Biosynthesis of unsaturated fatty acids**	arachidic acid<br>arachidonic acid<br>linoleic acid<br>oleic acid<br>palmitic acid<br>stearic acid	Amphetamine	**0.012**	**0.079**	**0.007**
		MDMA	**0.074**	0.553	0.172
		Mephedrone	**0.014**	**0.058**	**0.006**
**Linoleic acid metabolism**	12,13-EpOME<br>9,10-epoxyoctadecenoic acid<br>linoleic acid	Amphetamine	**0.021**	0.235	**0.031**
		MDMA	**0.007**	0.575	**0.028**
		Mephedrone	**0.023**	0.673	**0.080**
**Aminoacyl-tRNA biosynthesis**	L-alanine<br>L-glutamine<br>L-histidine<br>L-isoleucine/ L-leucine<br>L-phenylalanine<br>L-serine<br>L-tryptophan<br>L-tyrosine<br>L-valine	Amphetamine	0.7002	**0.026**	**0.092**
		MDMA	0.270	**0.012**	**0.022**
		Mephedrone	0.170	0.140	0.113
**Steroid hormone biosynthesis**	allopregnanolone<br>androsterone/ dehydroepiandrosterone/testosterone<br>androsterone glucuronide<br>dehydroepiandrosterone sulfate<br>cholesterol<br>hydroxycholesterol<br>dihydroxycholesterol<br>cortisol<br>dihydrocortisol/tetrahydrocortisone<br>cortisone	Amphetamine	0.998	**0.012**	**0.067**
		MDMA	1	0.142	0.420
		Mephedrone	0.991	**0.016**	**0.084**

**Primary bile acid biosynthesis**	chenodeoxycholic acid<br>chenodeoxycholic acid glycine conjugate<br>cholesterol<br>hydroxycholesterol<br>cholic acid<br>glycocholic acid<br>taurochenodeoxycholic acid	Amphetamine	0.67	**0.014**	**0.055**
		MDMA	0.960	**0.023**	**0.109**
		Mephedrone	0.904	0.890	0.979
**Tyrosine metabolism**	3,4-dihydroxymandelic acid<br>3-methoxytyramine<br>dopamine<br>epinephrine<br>L-tyrosine<br>maleylacetoacetic acid<br>norepinephrine<br>normetanephrine	Amphetamine	0.928	**0.023**	**0.106**
		MDMA	0.741	**0.012**	**0.052**
		Mephedrone	n.d.	n.d.	n.d.

R-Script for calculation of p-values using mixed-effect model statistics

```
#Model explained for one arbitrary feature
Datt3 <- returnData[["t3"]]
d7_3 <- datt3[,c(ncol(datt3)-1, ncol(datt3), ncol(datt3)-2, 7)]
colnames(d7_3)[4] <- "Int_t3"
# Extract the same feature for the baseline t0
datt0 <- returnData[["t0"]]
d7_0 <- datt0[,c(ncol(datt0)-1, ncol(datt0), ncol(datt0)-2, 7)]
colnames(d7_0)[4] <- "Int_t0"
# Join the two data frames
modelData <- left_join(d7_3, d7_0, by = c("Treatment", "Subj", "Week"))
modelData <- left_join(d7_7.5, d7_0, by = c("Treatment", "Subj", "Week"))
# Take the log of Int_t3 and Int_t0
# But, since we have zeros, we need to shift the data a bit in advance:
c <- 0.000001
modelData$log_Int_t3 <- log(modelData$Int_t3 + c)
modelData$log_Int_t7.5 <- log(modelData$Int_t7.5 + c)
modelData$log_Int_t0 <- log(modelData$Int_t0 + c)
head(modelData)
str(modelData)
# Model fit
model <- lmer(log_Int_t3 ~ log_Int_t0 + Treatment*Week + (1|Subj), data = modelData)
# Check assumptions
plot(model)
qqnorm(fitted(model))
qqnorm(ranef(model)$`Subj`[,1])
# Analysis of model output
anova(model)
summary(model)
spf <- interaction(modelData$Treatment, modelData$Week)
model2 <- lmer(log_Int_t3 ~ log_Int_t0 + spf + (1|Subj), data = modelData)
levels(spf)
# Build the linear hypothesis estimate
lc <- glht(model2, linfct = mcp(spf = rbind("Placebo - Amph" = c(-1/2,1/2,-1/2,1/2))))
```

```
slc <- summary(lc, test=adjusted("none"))

slc$test$pvalues

# p-value calculation

p.values <- mcmapply(function(i, data = datatp) {

d <- data[,c(ncol(data)-1, ncol(data), ncol(data)-2, i)]

colnames(d)[4] <- "Int"

d_0 <- datt0[,c(ncol(datt0)-1, ncol(datt0), ncol(datt0)-2, i)]

colnames(d_0)[4] <- "Int_t0"

modelData <- left_join(d, d_0, by = c("Treatment", "Subj", "Week"))

c <- 0.000001

modelData$log_Int <- log(modelData$Int + c)

modelData$log_Int_t0 <- log(modelData$Int_t0 + c)

spf <- interaction(modelData$Treatment, modelData$Week)

model_spf <- lmer(log_Int ~ log_Int_t0 + spf + (1|Subj), data = modelData)

lc <- glht(model_spf, linfct = mcp(spf = rbind("Placebo - Amph" = c(-1/2,1/2,-1/2,1/2))))

slc <- summary(lc, test=adjusted("none"))

pval <- slc$test$pvalues

names(pval) <- NULL

return(pval)

}, 1:(ncol(datatp)-3), mc.cores = ncor)

ordind <- order(p.values)

ord_p.values <- round(p.values[ordind],5)

# adjusted p.values by Benjamini-Hochberg

adj.p.values <- p.adjust(p.values, method = "fdr")

ord_adj.p.values <- round(adj.p.values[ordind],5)

feature.names <- colnames(datatp)[1:(ncol(datatp)-3)]

ord_feature.names <- feature.names[ordind]

res <- data.frame(feature = ord_feature.names, p.values = ord_p.values, adj.p.values =
ord_adj.p.values)

head(res,400)
```
